# Partitioning Transcript Variation in *Drosophila*: Abundance, Isoforms, and Alleles

**DOI:** 10.1534/g3.111.000596

**Published:** 2011-11-01

**Authors:** Yajie Yang, Rita M. Graze, Brandon M. Walts, Cecilia M. Lopez, Henry V. Baker, Marta L. Wayne, Sergey V. Nuzhdin, Lauren M. McIntyre

**Affiliations:** * Genetics Institute, University of Florida, Gainesville, FL 32610-3610; † Department of Molecular Genetics and Microbiology, University of Florida, Gainesville, FL 32610-0266; ‡ Department of Zoology, University of Florida, Gainesville, FL, 32611-8525; § Department of Statistics, University of Florida, Gainesville, FL 32611-8545; ** Molecular and Computational Biology, University of Southern California, Los Angeles, CA 90089-2910

**Keywords:** SNP chip

## Abstract

Multilevel analysis of transcription is facilitated by a new array design that includes modules for assessment of differential expression, isoform usage, and allelic imbalance in *Drosophila*. The ∼2.5 million feature chip incorporates a large number of controls, and it contains 18,769 3′ expression probe sets and 61,919 exon probe sets with probe sequences from *Drosophila melanogaster* and 60,118 SNP probe sets focused on *Drosophila simulans*. An experiment in *D. simulans* identified genes differentially expressed between males and females (34% in the 3′ expression module; 32% in the exon module). These proportions are consistent with previous reports, and there was good agreement (κ = 0.63) between the modules. Alternative isoform usage between the sexes was identified for 164 genes. The SNP module was verified with resequencing data. Concordance between resequencing and the chip design was greater than 99%. The design also proved apt in separating alleles based upon hybridization intensity. Concordance between the highest hybridization signals and the expected alleles in the genotype was greater than 96%. Intriguingly, allelic imbalance was detected for 37% of 6579 probe sets examined that contained heterozygous SNP loci. The large number of probes and multiple probe sets per gene in the 3′ expression and exon modules allows the array to be used in *D. melanogaster* and in closely related species. The SNP module can be used for allele specific expression and genotyping of *D. simulans*.

Gene expression analysis has proceeded from a primary focus on overall transcript level (Schena *et al.* 1995; Ross *et al.* 2000; Rifkin *et al.* 2003) to more sophisticated analyses, including those that examine expression of different isoforms (Johnson *et al.* 2003; Kwan *et al.* 2008) or individual alleles (Lo *et al.* 2003; Zhang *et al.* 2009). Commercial platforms exist for measuring 3′ expression or exon expression; however, there is not a single cost-effective platform for measuring expression at multiple levels. This article presents an array with three modules: 3′ expression, exon, and SNP probes for *Drosophila*.

In diploid organisms, expression from two, potentially different, copies of each gene contribute to transcript level and subsequent protein production. Unequal expression of these alleles is termed allelic imbalance (AI). AI is observed in model organisms and humans (*e.g.*, Lo *et al.* 2003; Guo *et al.* 2008; Graze *et al.* 2009; Zhang and Borevitz 2009). AI is a factor in predisposition to complex diseases (Meyer *et al.* 2008; de la Chapelle 2009) and contributes to phenotypic variation in human populations (Johnson *et al.* 2005; Pickrell *et al.* 2010). For example, AI is associated with the risk of developing breast cancer (Meyer *et al.* 2008) and colorectal cancer (de la Chapelle 2009).

AI has a genetic (as well as epigenetic) basis (*e.g.*, Pastinen *et al.* 2004; Serre *et al.* 2008; Wang *et al.* 2008; Verlaan *et al.* 2009). Exciting new developments in the study of complex diseases revealed regulatory polymorphisms contributing to the evolution of gene regulation (*e.g.*, Emerson *et al.* 2010). Whole-genome associations of gene expression and phenotype identify the genetic basis of disease and other important phenotypic variation (Stranger *et al.* 2007; Nica and Dermitzakis 2008; Nica *et al.* 2010). AI identifies causal *cis* regulatory variants (Wittkopp *et al.* 2004). Allele-specific association studies advance these analyses and increase scientific knowledge of the regulatory process (Rockman and Kruglyak 2006; Serre *et al.* 2008; Stamatoyannopoulos 2004). Analysis of AI is an important next step in identifying the genetic basis of expression differences.

AI has been assayed with pyrosequencing (Ahmadian *et al.* 2000; Wittkopp *et al.* 2004), targeted SNP typing arrays (*e.g.*, Serre *et al.* 2008), high-density array designs (*e.g.*, Zhang and Borevitz 2009), RNA-Seq–based methods (Zhang *et al.* 2009 ; McManus *et al.* 2010; Pickrell *et al.* 2010), and smaller-scale methods, such as allele-specific qPCR (Szabó and Mann 1995).

This article presents a custom array for measuring 3′ expression, exon expression (and thus alternative splicing), and AI. The array has been designed for *Drosophila* on an Affymetrix platform (UFL Custom Dros_snpa520726F Array Format: 49-7875; available for purchase from Affymetrix). The use of a single platform is cost effective, and statistical analysis is simplified by the single hybridization. We designed 60,118 *D. simulans* SNP probe sets from previously reported SNP variants (Benson *et al.* 2005; Begun *et al.* 2007). In total, these probe sets allow AI to be assessed for 11,929 genes [79% of 15,107 genes in FlyBase R5.11 (August 2008)], with the majority of genes represented by multiple SNP probe sets. The SNP module is complemented by two additional modules: one that measures 3′ expression and another that analyzes exon-level expression concurrently with allele specific expression (ASE). Experiments show an amount of sex bias (34% of 18,769 probe sets), alternative exon usage (164 genes), and AI (37% of 6579 probe sets within a species) consistent with previous reports on other platforms (McIntyre *et al.* 2006; Wayne *et al.* 2007; Telonis-Scott *et al.* 2008; Fontanillas *et al.* 2010).

## Materials and Methods

### Chip design

**Figure 1:**
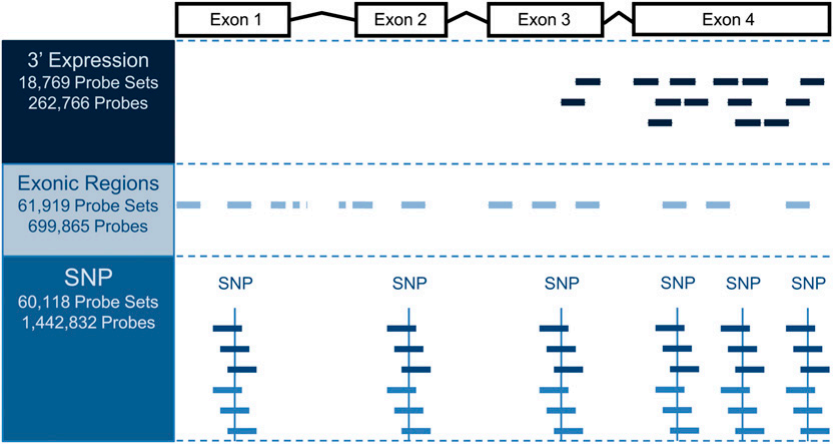
Probe design. A total of 2,424,414 probes were printed on the chip. They are of four types: SNP probes (*n =* 1,442,832; 60,118 probe sets), 3′ expression probes (*n =* 262,766; 18,769 probe sets), exon probes (*n =* 699,865; 61,919 probe sets), and control probes (not shown). The 3′ expression probes consist of all perfect-match (PM) probes from the Affymetrix GeneChip *Drosophila* Genome 2.0 array. An example of a 3′ expression probe set is shown in navy. The exon probes consist of all Affymetrix *Drosophila* Tiling 2.0 Array probes that map uniquely to exonic regions annotated in FlyBase R5.11 (August 2008). Exon probe sets within the example gene are shown in light blue. SNP probes are custom made. The SNP probes corresponding to a single SNP site base are shown in dark blue (matching the forward strand) and blue (matching the reverse strand).

The chip has 2,424,414 informative features, covering four types of probes: SNP probes (*n =* 1,442,832; 60,118 probe sets); 3′ expression probes (*n =* 262,766; 18,769 probe sets); exon probes (*n =* 699,865; 61,919 probe sets); and control probes (16,943 GC band controls; 2008 hybridization and labeling controls; Figure 1). The 3′ expression probes consist of all perfect-match (PM) probes from the Affymetrix GeneChip *Drosophila* Genome 2.0 array (900531, 900532, and 900533). The exon probe sets provide measurements of expression from each individual exon, allowing controls for signal fluctuation caused by 5′ bias in expression assays, as well as measurement of alternative exon usage. The exon probes consist of all Affymetrix *Drosophila* Tiling 2.0 Array (901021) probes that map uniquely to exonic regions (FlyBase R5.11 August 2008) at the time of chip design. Overlapping exons with alternative start/end sites in the same genomic region were combined into a single exonic region. The majority of exonic regions contain a single exon. (For simplicity, exonic regions are referred to simply as exons throughout this article.) Each exon corresponds to a unique probe set. The 3′ expression probes and exon probes on this custom chip were designed by Affymetrix from *D. melanogaster* sequences. The probe sets have been used for other *Drosophila* species (*i.e.*, Kopp *et al.* 2008; Graze *et al.* 2009; Dworkin and Jones 2009; Lu *et al.* 2010). Using these probe sets allows direct comparisons to existing literature and straightforward quality control. As each probe set has multiple probes, the impact of divergence is likely to be minimal on summary measures of expression. However, investigators comparing among species should consider filtering individual probes. The SNP module was designed for estimating AI. There were three main steps in this design: SNP identification, SNP quality assessment, and probe selection.

#### SNP identification:

Alignment sets were created from multiple sequence sources, including FlyBase R5.4 exons (*n* = 68,536), six *D. simulans* strain genomes for *Drosophila* Population Genomics Project (DPGP, http://www.dpgp.org, Begun *et al.* 2007), and all *D. simulans* sequences (343,420) from GenBank (Benson *et al.* 2005) that were not annotated as “whole genome.” In DPGP, *D. simulans* genomes, except for the heterochromatic regions, were assembled against the FlyBase R4.2 *D. melanogaster* genome. Exons from FlyBase R5.4 were BLAST (Altschul *et al.* 1990) aligned to the DPGP genomes and GenBank sequences. There were 325 exons with only GenBank sequence, 62,161 with only DPGP sequence, 3163 with both GenBank and DPGP sequence, and 2887 for which no sequence was available. For each FlyBase R5.4 exon, its genome location in *D. melanogaster* R4.2 genome was determined by BLAST exons that matched more than one location; those located on chromosomes four or U were excluded (*n* = 1912). All matching sequences for each exon were aligned using ClustalW (Thompson *et al.* 1994) to create a multiple sequence alignment at the exon’s genome position. All SNPs, regardless of location in the exon, were identified from the multiple sequence alignment.

#### SNP quality assessment:

**Figure 2:**
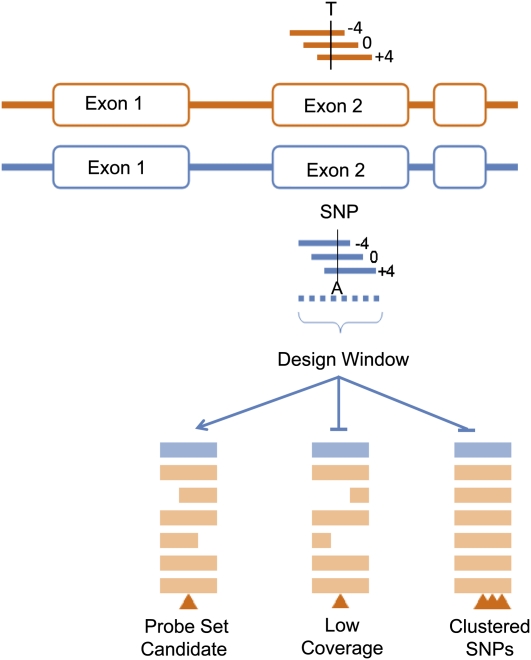
SNP probe design windows. For each SNP site, there are four sets of probes, one for each SNP site base. The SNP base is designed at three different positions of the probes: middle, shifted four bases upstream, or shifted four bases downstream. Each SNP probe set contains 24 probes, which can be classified based on alleles as PM1 (*n* = 6), PM2 (*n* = 6), or MM (*n* = 12), for a total of 24 probes per probe set. A SNP probe set has a 35-base design window, with sequences of −17 bases upstream and 17 bases downstream from the SNP. If there were fewer than five sequences supporting a SNP, the SNP was discarded. If more than one SNP occurred in the design window, then the alignment was considered suspect, and the SNP was not included among those printed on the array. Only biallelic SNPs that were unique in their design window and supported by five or greater sequences in the multiple alignment were considered.

A design window, which consisted of −17 bases upstream and 17 bases downstream from each SNP (Figure 2) was the basis of SNP quality assessment. A SNP locus supported by fewer than five sequences was discarded. SNPs were also discarded when the design window mapped to multiple places in the genome or when more than one SNP occurred in the design window. These criteria identified 589,915 SNPs, of which 196,345 were biallelic. Only biallelic SNPs were considered further. There were 558 exons for which SNP data were identified from GenBank alone, 51,418 exons for which SNP data were identified from DPGP alone, and 2992 identified from both, resulting in a total of 54,968 exons with SNPs present; in other words, 81% coverage of the entire FlyBase R5.4 transcriptome (68,536 exons).

#### Probe selection:

For each SNP, 24 probes were designed, with the SNP at the 0, +4, and −4 positions from the probe center, for the forward and reverse strands, and with each possible base (A, C, G, and T) at the SNP site. Probe hybridization quality was predicted by an Affymetrix internal scoring algorithm that takes into account Tm, secondary structure, and previous empirical observations. If a probe contained a homopolymer run or could not be synthesized or if one third or more probes had poor predicted hybridization, the probe set was eliminated. For genes with seven or fewer SNPs, all SNPs were selected. If a gene had more than seven SNPs, additional probe sets were selected at random (*n* = 610) to fill the chip.

**Figure 3:**
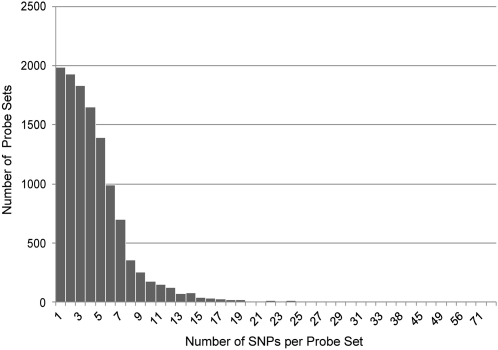
Distribution of SNP probe sets per gene. A total of 60,118 probe sets, representing 11,929 genes, were selected for the SNP module for the custom chip. The number of genes (Y axis) with a given number of corresponding SNP probe sets (X axis) is shown. Most genes are represented on the array by one to five SNP probe sets.

In sum, 60,118 custom SNP probe sets representing 11,929 genes (Figure 3) were included on the chip. The mean number of probe sets per gene was 4.4. The majority (8013 genes) had more than 3 probe sets. The chip library files are available at http://bioinformatics.ufl.edu/McIntyre_Lab/ASE. Probe sequences and chip annotation can be found at Gene Expression Omnibus (GEO) using accession ID GPL11273.

### Verifying the experiment fly materials

#### Experimental design:

Two different isogenic strains of *D. simulans*, *st e* and C167.4, and their male and female progeny were used as the basis for the verification study. Three replicates of RNA from female and male progeny of the cross *st e* × C167.4 were assayed for six RNA samples. In addition, DNA was used as a control for estimating AI (Wittkopp *et al.* 2004; Wittkopp *et al.* 2008; Degner *et al.* 2009; McManus *et al.* 2010). Three replicate gDNA samples were prepared for female *st e*, female C167.4, and the female F_1_ progeny of the cross *st e* × C167.4, for nine gDNA samples.

#### Sample collection:

Flies were reared in incubators (25**°**, 12:12 hr light/dark cycle) on a standard dextrose medium. Isogenic strains of *D. simulans* (C167.4, BDSC 4736; *st e* isogenic, DSSC 14021-0251.041 inbred >20 generations) were used. For each of three cross genotypes (C167.4, *st e*, and C167.4 × *st e*), 20 virgin females were crossed to 5 males. Female and male progeny were collected on consecutive days (under CO_2_) and aged from 5 to 7.5 days in single sex vials. Flies were then flash frozen in liquid nitrogen (without anesthesia) in a 2.5 hr window (4–6:30 pm). For RNA samples, two sets of 20 flies (subsamples) were collected for each replicate from multiple cross vials. No vials were used for more than one replicate.

#### Sample processing:

Flies were freeze dried at −20° overnight prior to homogenization. Dried flies were ground to a fine powder using a GenoGrinder (maximum, 3 min, repeated twice). Trizol (1 ml) was added to each homogenized sample and mixed thoroughly in the GenoGrinder (maximum, 3 min). Samples were transferred to a new tube, 1 μl linear acrylamide was added to each, and then samples were incubated at room temperature for 5 min. RNA was extracted using a standard Trizol extraction protocol: phase separation using 0.1 vol BCP, RNA precipitation with isopropanol, 70% ethanol wash, and resuspension in 80 μl DEPC H_2_O. Concentration was measured using a NanoDrop, and up to 30 μg RNA per sample was treated with DNase I in 100 μl reaction volumes for 30 min at 37° (reaction mix: 4 U Cloned DNase I TaKaRa 2220A, 80 U Promega Recombinant RNasin N2515, in 1× TaKaRa Cloned DNase I Buffer II). Samples were cleaned prior to concentration using the Qiagen RNeasy Mini Kit (Cat. #74104) following the manufacturer’s standard protocol with 30 μl DEPC H_2_O elutions (run through the column twice). RNA quality was examined using BioAnalyzer RNA 6000 Nano chips, and all samples were found to be of good quality. Genomic DNA was isolated from 35 to 40 flash frozen females using the AllPrep Mini Kit (Qiagen) following standard manufacturer’s protocols. Samples were concentrated by standard ethanol precipitation and resuspended in 31 μl DEPC H_2_O.

#### Fragmentation, labeling, and array hybridization:

Target materials were prepared for array hybridization using the recommended Affymetrix kits following the no amplification protocol of GeneChip© WT Double-Stranded Target Assay Manual (DNA samples started from Procedure D forward) for single Tiling Arrays. Briefly, 10 μg of total RNA was concentrated to 8 μl in DEPC H_2_O followed by first- and second-strand cDNA synthesis using the WT Double-Stranded DNA Synthesis Kit (P/N 900813). Per the GeneChip Sample Cleanup Manual (P/N 900371), 7.5 μg of dsDNA was fragmented. For each DNA sample, 7.5 μg of gDNA was fragmented to between 25 and 200 bp with 0.02 U/μg DNase I (Takara Cloned DNase I, 2 U/μl) in a 40 μl reaction with 4 μl reaction buffer (10× reaction buffer: 100 mM Tris-acetate, 100 mM magnesium acetate, 100 mM potassium acetate) and 0.8 μl BSA (10 mg/ml). Reactions were incubated 16 minutes at 37° and heat killed at 99° for 15 minutes. Fragment size was checked by agarose gel electrophoresis. Fragmented cDNA and gDNA targets were labeled using WT Double-Stranded DNA Terminal Labeling Kit (P/N 900812). The prepared target samples were hybridized using the Hybridization, Wash, and Stain Kit (P/N 900720) following the manufacturer’s protocol (FS450_0001) for the Fluidics Station 450 with protocol. Arrays were scanned using an Affymetrix 7G scanner. The GEO accession for the array data is GSE31750.

### Signal quantification

Signals were extracted from the scans using the apt-cel-extract program of the Affymetrix Power Tools (version 1.10.2) suite. GC bin control probes provide an estimate of nonspecific hybridization (Affymetrix 2005) and help to assess the overall quality of the hybridization. A GC bin control is a standard Affymetrix control based upon the number of G/C bases (from 3 to 24) in the 25 mer probe. None of the GC bin control probes align to the *D. melanogaster* or *D. simulans* reference genomes. Individual probes were classified according to their GC content and matched to the corresponding GC bin controls. A probe was considered detected when signal strength was higher than the median intensity of the corresponding GC band controls. Detection above background (DABG) was calculated at the individual probe level. The overall intensity of the array was evaluated at the individual probe level. To correct for the background noise and to normalize the probe signals, each probe was classified into a GC bin and the 5 percentile signal for that GC bin was subtracted from the probe signal. Y_i_, the signal for probe set *i*, is estimated as: $Y_{i}=ln\left(\sum_{j}\left(X_{ij}-GC_{j}\right)/N_{i}+100\right)$. X_ij_ is the intensity for probe *j* in probe set *i* and GC_j_ is the average intensity for control probes in the corresponding GC bin. N_i_ is the number of probes in probe set *i*. Chip verification was analyzed first for the overall hybridization quality, then for each module on the chip (3′ expression module, exon module, and SNP module).

### General quality control

The distribution of the overall signal across all modules was compared using kernel density estimates for each slide separately (Silverman 1986), with the goal of identifying any slide with an unusual distribution. Similar marginal distributions of kernel density would be expected for one sample type. Principle component analysis (PCA) (Johnson and Wichern 1992) was carried out to determine whether there was any pattern or grouping to the data.

To verify the veracity of probe set estimates of 3′ expression, we compared the signal from probe sets for the well-known sex-biased genes (Bownes 1994; Wolfner 1997): Yp (Yp1, Yp2, Yp3) and Acp (Acp29AB, Acp32CD, Acp36DE, Acp53C14a, Acp53C14b, Acp53C14c, Acp62F, Acp76A). Consistency of estimation of gene expression across modules was also examined using Bland-Altman plots (Bland and Altman 1986; Bland and Altman 1988; Dudoit *et al.* 2002; McIntyre *et al.* 2011), in which the exon module and SNP module were plotted against the 3′ expression module.

The feature size of this array is smaller (5 micron) than is the Affymetrix GeneChip *Drosophila* Genome 2.0 array (11 micron). Although the PM probes are identical for these two chips, feature size may have an impact on differential expression (Dandy *et al.* 2007; Ammar *et al.* 2009). This raises the concern of potential loss of sensitivity. To evaluate the performance of the 3′ expression and exon modules, we compared expression for RNA samples between the two sexes of the F_1_ progeny. Sex-biased expression is well described for *Drosophila* (Bownes 1994; Jin *et al.* 2001; Parisi *et al.* 2003; Ranz *et al.* 2003; McIntyre *et al.* 2006; Telonis-Scott *et al.* 2008).

The fixed effects model,

$$Y_{ij}=\mu +{\text{s}}_{i}+\varepsilon_{ij}$$ (1)

was fit for each probe set in the 3′expression and exon modules, where Y_ij_ is the signal for probe set *i*, sample *j* is as described above, μ is the overall mean, s_i_ is the fixed effect of sex, and ε_ij_ is the random error. The null hypothesis that male and female sexes had equal expression levels was tested using an F-test (Neter *et al.* 1990). All probes in a given probe set were used. As only one genotype is considered, any polymorphisms between the genotype used and the probe will be the same between the two sexes of the same genotype. Results were corrected for multiple testing using False Discovery Rate (FDR) (Benjamini and Hochberg 1995; Verhoeven *et al.* 2005). Where multiple probe sets matched the same genes, the agreement between the exon probe sets and the 3′ expression probe sets were examined for agreement in detecting sex bias using Kappa statistics (Fleiss 1981) and McNemar’s test (Johnson and Wichern 1992).

There is a sex bias in isoform usage in *Drosophila* (Kwan *et al.* 2008; Telonis-Scott *et al.* 2008; McIntyre *et al.* 2006). The use of alternative transcript isoforms between the two sexes can be detected from the measurements taken by the exon module. A probe set represents a constitutive exon for a gene (included in all annotated isoforms) or an alternative exon (included in a subset of known isoforms). Inferences can become ambiguous when probe set annotations correspond to exon regions located in overlapping regions of multiple gene models. Probe sets mapping to more than one gene or to the ambiguous regions of overlapping exons were excluded from analysis (*n* = 2611). The model

$$Y_{ij}=\mu +x_{i}+s_{j}+xs_{ij}+\varepsilon_{ij}$$ (2)

where *x* is the fixed effect of exon type and *s* is the fixed effect of sex was fit. Probe sets from multiple constitutive exons were grouped as one exon type, whereas probe sets representing alternative exons were each considered a different exon type. The variance was estimated separately for each sex. The significance of the interaction (a test for alternative isoform usage McIntyre *et al.* 2006) was tested using an F test, followed by FDR correction.

#### SNP calling and genotyping accuracy:

By design, there are DPGP/GenBank sequences for all 60,118 probe sets in the SNP set, from which biallelic SNPs were defined and used for the chip design. SNP alleles were verified using Illumina genome resequencing data for the C167.4 and *st e* strains (GEO accession SRP005952) of *D. simulans*. *D. simulans* C167.4 sequence data were obtained from male head RNA libraries sequenced on multiple lanes with Illumina paired end procedures and chemistry (Celniker *et al.* 2009; McIntyre *et al.* 2011). *D. simulans st e* sequences were from genomic DNA extracted from adult *st e D. simulans* females. Average coverage was 30×. Reads were aligned to updated reference genomes (R. Graze *et al.*, unpublished data) using Bowtie (Langmead *et al.* 2009) and LAST (Frith *et al.* 2010). Alignments were converted to pileup format using SAMtools (Li *et al.* 2009). SNP bases were identified from the pileup alignments and compared with alleles identified from DPGP/GenBank. The bases identified from C167.4 resequencing were also compared with the DPGP genome sequences for the C167.4 strain.

### Analysis of AI

To verify the chip’s capacity to identify differences in AI, a subset of SNP probe sets unambiguous for the two alleles from the design and confirmed by the C167.4 resequencing and *st e* resequencing (where the F_1_ is heterozygous) were selected. The model,

$$Y_{ijk}=\mu +s_{i}+t_{j}+\varepsilon_{ijk}$$ (3)

was fit for probe sets in this subset using the nine F_1_ arrays (six RNA, three DNA). Y_ij_ is the normalized signal value for the *i^th^* sex, *j^th^* treatment, and the *k^th^* replicate. The treatment groups were defined by combinations of nucleic acid (DNA/RNA) and allele (PM1, PM2, and MM) for a total of *j* = 1…6 levels and *k* = 1…3 replicates. AI was examined by testing the difference in hybridization intensity between PM1 and PM2 in the RNA, compared with the difference in the DNA. An F test for this contrast was performed, and the result was corrected for multiple testing using FDR.

The power of detection of AI effects may differ between the sexes due to sex bias in gene expression. There may also be sex-specific differences in AI. Both phenomena would result in a difference in detection of AI between the sexes. Unfortunately, the power for the test of an interaction was low. The AI was also analyzed considering the female and male data separately so that any differences between the sexes in the results might be apparent.

## Results

### Quality control

**Figure 4:**
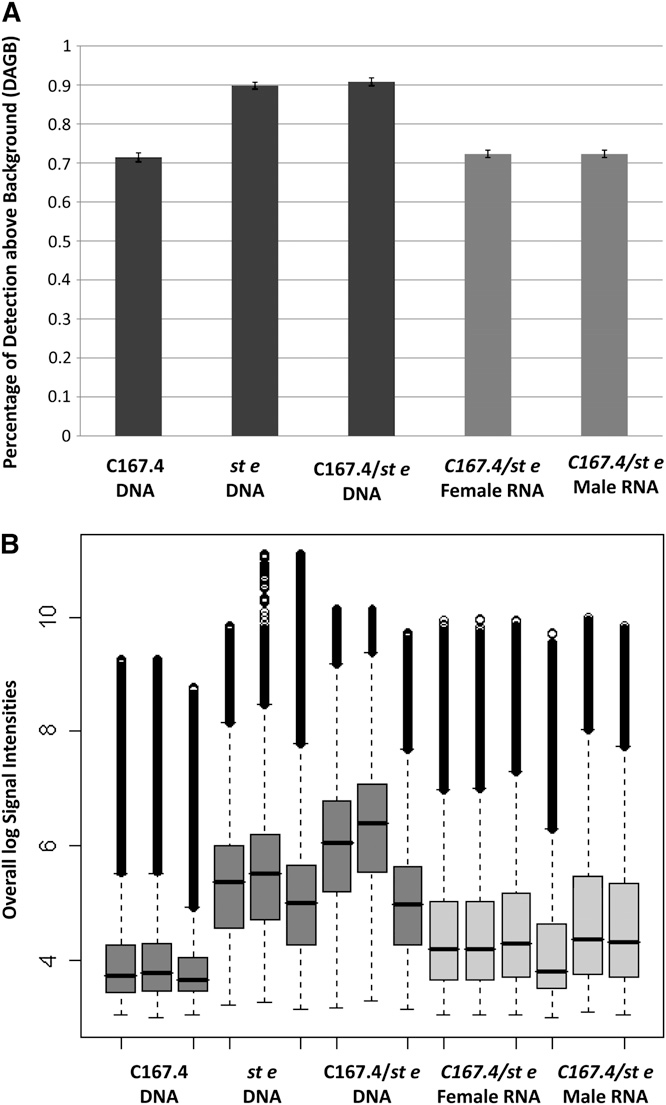
Quality control analyses of the normalized data. (A) The proportion of probes detected above background (DABG) is reported for all probes sets of each sample: C167.4 parental DNA, *st e* parental DNA, and DNA and RNA of the F_1_ genotype. DNA samples are shown in dark gray. RNA samples are shown in light gray. The Y axis is the overall percentage of DABG. Probes were classified according to their GC content and matched to the GC band controls of the corresponding %GC bin. A probe was considered detected when signal strength was higher than the median intensity of the corresponding GC band controls. The three C167.4 parental DNA hybridizations had lower DABG compared with the other two genotypes of DNA samples. (B) Box plot for probe intensity classified by genotype and nucleic acid. DNA samples are shown in dark gray. RNA samples are shown in light gray. The Y axis is the normalized signal. Probes were classified according to their GC content and matched to the GC band controls of the corresponding %GC bin. The five percentile signal for that GC bin was subtracted from the probe for background correction. The corrected signals were then log-transformed. The three C167.4 parental DNA hybridizations had overall weaker signals.

**Figure 5:**
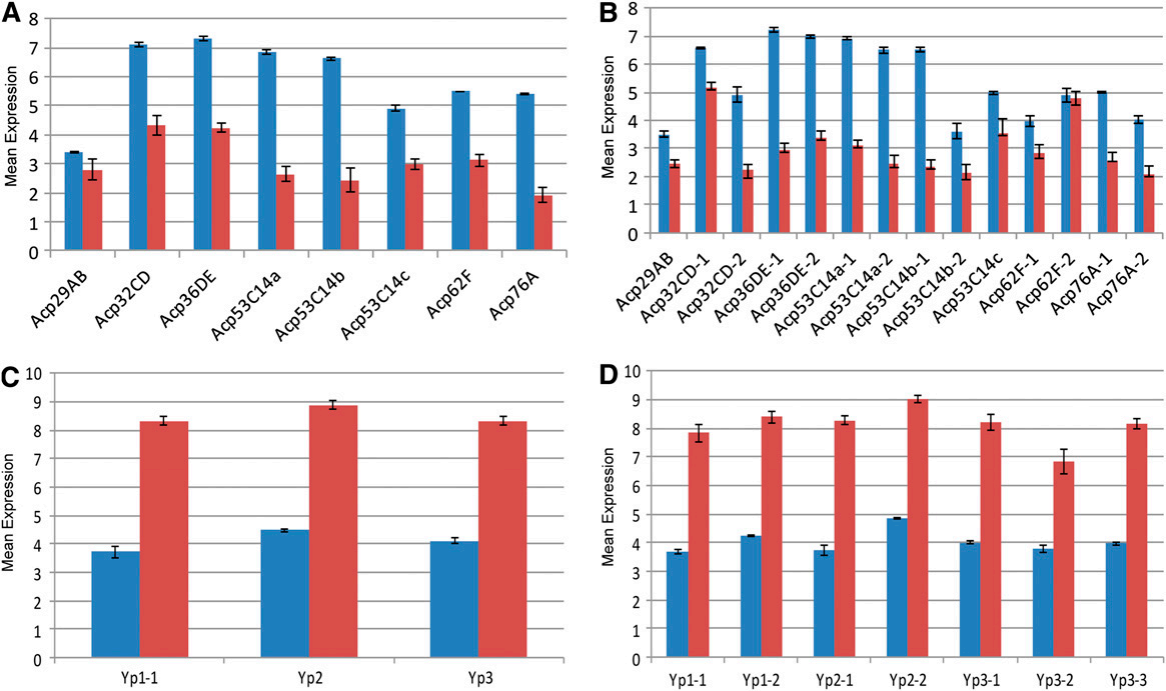
Expression for known sex-specific genes in female and male RNA samples. The Y axis is the normalized signal. A value around or lower than 3 is close to the background intensity and, therefore, should be considered as not detected. Female samples are shown in red. Male samples are shown in blue. (A) The mean signals of all probe sets for each Acp gene. (B) The expression of individual probe sets designed for Acp genes. (C) The mean signals of all probe sets for each Yp gene. (D) The expression of an individual probe set that was designed for Yp genes. The directions of sex bias are as expected (Acps are male-specific genes, and Yps are female-specific genes). Individual probe sets for the same gene behave consistently.

Quality control evaluations showed that the three C167.4 parental DNA hybridizations had overall weaker signals and that the kernel density distribution was markedly different from all of the other chips. The DABG was only 70% for these hybridizations (Figure 4A) compared with ∼90% for other DNA samples. The remaining chips showed no obvious problems with hybridization. All modules (3′ expression, exon, and SNP) had similar proportions of probes detected above the median of the GC band control signals (Table 1). The proportions were ∼72% for RNA samples and ∼90% for DNA samples. The distribution of signal values across all modules was similar for all RNA hybridizations and differed from DNA hybridizations (Figure 4B). PCA identified no other hybridization anomalies. Both sexes had similar hybridization patterns (supporting information, Figure S1). The normalized signal intensities of the exon module probe sets and expression module probe sets for Acp and Yp genes gave the expected results (Figure 5). The average intensity for each gene was consistent between modules (Figure S2).

**Table 1:** Proportions of probes detected above the median GC band control

Genotype	Nucleic acid	Sex	Overall DABG	Exon module DABG	Expression module DABG	SNP module DABG
C167.4	DNA	Female	0.714435	0.744564	0.750768	0.6968
*st e*	DNA	Female	0.898116	0.897156	0.919405	0.899683
C167.4/*st e*	DNA	Female	0.908025	0.903021	0.915376	0.914164
C167.4/*st e*	RNA	Female	0.722919	0.791827	0.761107	0.770503
C167.4/*st e*	RNA	Male	0.722919	0.812318	0.79766	0.791968

The average proportion of probes detected above background (DABG) for genotype, nucleic acid, and sex. An individual probe was detected when signal strength was higher than the median intensity of the corresponding GC band control. Average DABG was calculated for each individual module and for the overall slide. The individual modules of expression, exon, and SNP, as well as the entire slide, had similar proportions of probes detected above the median GC band control. The distribution of signal values across all modules was similar for all RNA hybridizations and differed from DNA hybridizations. The three C167.4 parental DNA hybridizations had lower DABG compared with the other two genotypes of DNA samples.

To test the smaller format features of the 3′ expression probe sets, we analyzed the effect of sex on expression level. Previous studies in *Drosophila* (Bownes 1994; Jin *et al.* 2001; Parisi *et al.* 2003; Ranz *et al.* 2003; McIntyre *et al.* 2006; Telonis-Scott *et al.* 2008) have all found sex bias in overall expression. Analysis of differential expression on the 3′ expression module revealed a strong effect of sex on gene expression. Three different significance levels (FDR < 0.05, FDR < 0.1, and FDR < 0.2) are reported (Table 2). Raw *P* values and adjusted FDR *P* values are in Table S1. Among all sex-biased probe sets at FDR < 0.1, 2216 had higher expression in males and 4140 had higher expression in females. Sex bias of expression was similarly analyzed for exon probe sets corresponding to single exons that exist in all transcripts of a gene (constitutive) (*n* = 47,122; Table 2; Table S3). The results for exon probe sets were compared with the 3′ expression probe sets corresponding to the same genes. There were 2091 genes with probe sets corresponding to exons contained in all transcripts, which could be easily matched to a single probe set in the 3′ expression module. The majority of genes showed similar sex bias between the 3′ expression and exon module, and simple agreement was high (1720, 82%). The Kappa statistic was 0.63 between the two modules, indicating good agreement. There was no apparent asymmetry in detection, with 106 genes detected by the exon module alone and 265 genes detected by the 3′ expression module alone. The McNemar’s test statistics was 68.1429 with *P* value smaller than 0.0001. There were 164 genes that showed a significant interaction between exon type and sex; these were considered as showing putative isoform-specific sex bias (FDR < 0.1; Table S2).

**Table 2:** Tests for the effect of sex from the 3′ expression and exon probe modules at multiple FDR levels

	FDR < 0.05	FDR < 0.1	FDR < 0.2
3′ Expression module (*n* = 18,769)	3,607 (19.22%)	6,356 (33.86%)	9,574 (51.01%)
Exon module (*n* = 47,122)	6,201 (13.16%)	15,241 (32.34%)	26,226 (55.66%)

The probe sets in the 3′ expression module and the exon module were tested for sex-biased expression. The number of significant probe sets and the percentage of significant probe sets over all probe sets within a module are shown. Results from different significance thresholds (FDR < 0.05, 0.1, and 0.2) all indicate a strong sex effect measured by both the expression module and the exon module.

### Verifying the SNP module

The *D. simulans* sequences used to design the SNP probes were sequenced as part of the DPGP. The sequencing strategy of DPGP was to sequence one line at ∼4× coverage and six additional lines (including C167.4) at 1×. The confidence for SNP calls, therefore, varied depending on quality and depth. There were 60,118 probe sets, and of these, 42,978 had SNP base information for C167.4 from DPGP, 35,379 had additional data available from Illumina sequencing of C167.4 RNA, and 49,758 had additional data from Illumina sequencing of *st e* DNA.

The concordance of C167.4 SNP base calls from the ∼1× DPGP C167.4 strain genome sequence used for the chip design and the resequencing was 66.72%, significantly larger than expected by chance. This rate did not affect the quality of the SNP probe sets, as C167.4 was only one of the seven lines in DPGP that were used for our chip design. The concordance between the resequencing bases and the alleles used in the design was impressive. The C167.4 RNA-Seq base agreed with one of the two alleles identified in the design 99.58% of the time. Agreement between the *st e* DNA-Seq bases and the two alleles in the design was 99.75%.

Next, the concordance between the resequencing and hybridization was examined by comparing resequencing SNP base calls to the probe bases ranked by the strength of their hybridization signals. The comparison was carried out separately for cases where the resequencing bases were the same for C167.4 and *st e* (homozygous F_1_) or where they were different (heterozygous F_1_). For *n* = 13,573 SNP probe sets homozygous at the SNP site in the C167.4/*st e* genotype, the ranked hybridization intensities of probes corresponding to each base (within a given SNP probe set) was compared with the predicted genotype at the SNP base (Table 3). A SNP probe set was included in this comparison when the common SNP allele call for C167.4 and *st e* strains corresponded to one of the alleles in the chip design, and the base was also the same in the DPGP sequence for the C167.4 strain. For example, if the SNP allele in both strains is A (with respect to the forward strand), probes corresponding to targets with A at the SNP position are expected to show increased hybridization intensity relative to the signal for the T, G, and C probes for all genotypes tested. The percentage of probe sets where the probes corresponding to the target SNP allele show the highest intensity is reported overall and separately for each base (Table 3). The concordance between the resequencing SNP and the C167.4 DNA arrays was significantly lower than other arrays, likely caused by the weaker signal intensity of the C167.4 DNA arrays. Similarly, the ranked hybridization intensities of probes corresponding to each base (within a given SNP probe set) was compared with the predicted genotype at the SNP base for *n* = 2769 SNP probe sets heterozygous at the SNP site in the C167.4/*st e* genotype (Table S4). Our observed concordance is striking, given the small number of genotypes used in this experiment. A previous study (Borevitz *et al.* 2007) using arrays for detecting sequence polymorphisms reported a similar error rate. Larger experiments with more samples can reduce the error rate (Edenberg *et al.* 2005; Rabbee and Speed 2006). In comparison, a short-read sequencing experiment requires more than 200 reads unambiguously mapped to the gene for each gene to achieve a similar result (Fontanillas *et al.* 2010).

**Table 3:** Rank of hybridization signal corresponds to the expectation based on sequence information (homozygous genotypes)

SNP allele	Hybridized arrays			
	C167.4 DNA	*st e* DNA	C167.4/*st e* DNA	C167.4/*st e* RNA
Overall	71.47%	91.54%	86.95%	90.09%
A	59.98%	84.82%	79.90%	83.11%
C	77.12%	94.35%	90.23%	93.15%
G	77.07%	95.26%	90.30%	93.30%
T	59.94%	84.90%	80.44%	84.23%

Considered were probe sets where 1) SNP allele calls for the C167.4 and *st e* strains correspond to the PM1 and PM2 alleles in the chip design; 2) the C167.4 base is concordant between the resequencing data and the DPGP sequence for the C167.4 strain; and 3) the C167.4/*st e* genotype is homozygous for the SNP site (*n* = 13,573). The signal from each base was estimated as the average of the probes representing that base. For each probe set, the four bases were ranked according to signal, and the base with the greatest hybridization signal was compared with the known base. The percentage of probe sets for which the base corresponding to the top-ranked hybridization intensity was the known allele was calculated. Percentages are reported considering all SNP bases and separately for A, C, G, and T alleles. The concordance between the resequencing SNP and the C167.4 DNA arrays was significantly lower than other arrays, likely due to the weaker signal intensity of the C167.4 DNA arrays.

**Figure 6:**
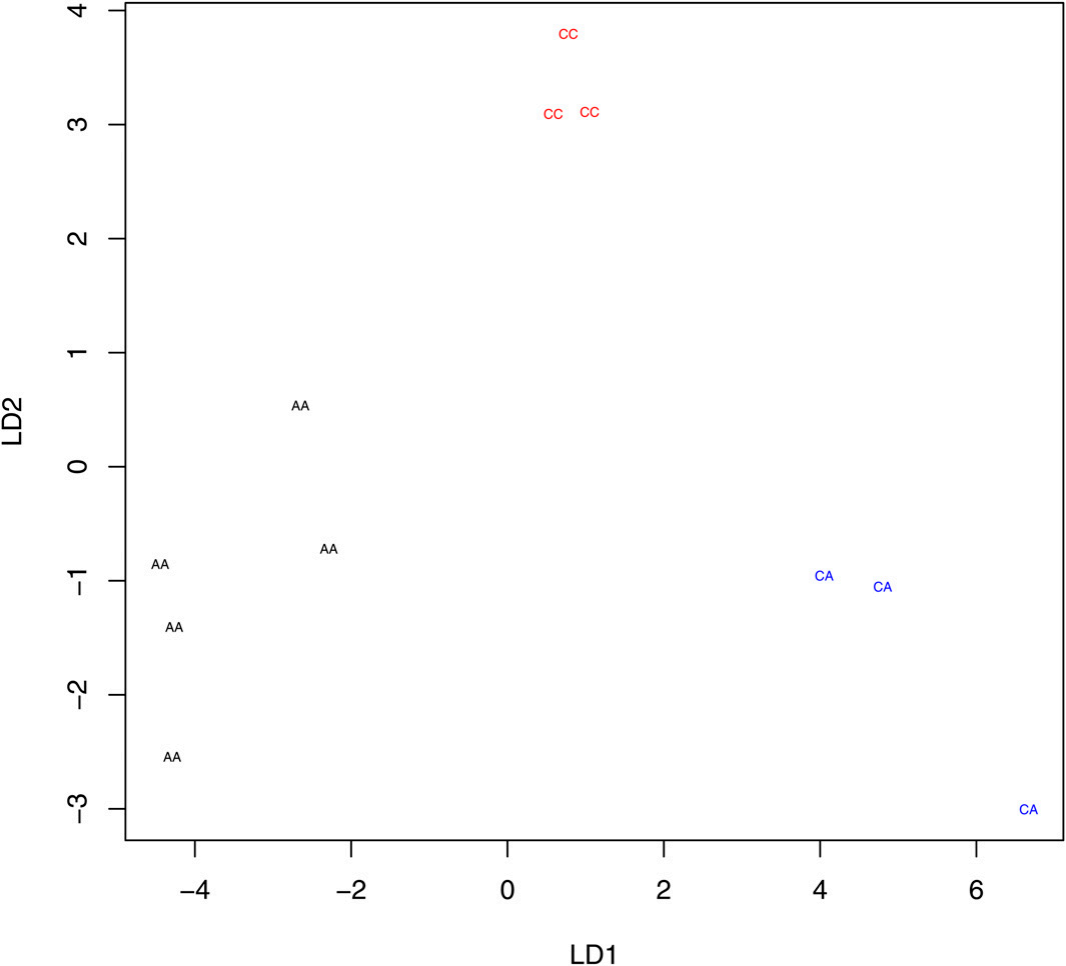
Linear discriminant plots of three genotypes: AA, AC, and CC. Different genotypes had hybridization patterns that are visually separable by linear discriminant (LD) analysis. Each genotype is a different color.

The hybridization signals were compared with the two alleles (PM1 and PM2) used in the design. The concordances between the two highest ranked probe bases and the PM1 and PM2 bases were high: 96.22% for C167.4 DNA chips and 98.77% for *st e* DNA chips. For hybrid F_1_’s, the concordance percentage was 98.62% for DNA chips and 97.53% for RNA chips. To determine whether the pattern of hybridization could be used to predict genotype, we performed a linear discriminate analysis (Johnson and Wichern 1992) for several arbitrarily selected probe sets heterozygous for the SNP base in the F_1_ hybrid. All that we examined showed visual separation of the expression patterns (Figure 6). These comparisons confirmed that the hybridizations performed as expected.

### AI analysis

There were 33,914 unambiguous probe sets with sequence information for both the *st e* and C167.4 resequencing experiments at the SNP site. The SNP base was the same for the parental two lines for 74.78% of these probe sets, which were classified as homozygous for the F_1_ genotypes (*n* = 25,362). The rest of the probe sets (8552) had heterozygous F_1_ genotypes. The 6579 autosomal probe sets were analyzed for AI on the combined data from both sexes and for each sex separately (Table 4; Table S5).

**Table 4:** Allele imbalance overall and separated by sex (*n* = 6,579)

	FDR < 0.05	FDR < 0.1	FDR < 0.2
Both sexes	2013	2453	3004
Male	1657	2028	2497
Female	923	1384	1899

AI was tested for male and female samples alone and combined. Results from different significance cutoffs (FDR < 0.05, 0.1, and 0.2) are shown. The numbers of probe sets with a significant AI effect are reported. There are large proportions of genes that have significant AI in male samples, female samples, and combined (male/female) samples.

## Conclusion

Our custom platform performs similarly to previous array platforms with a larger feature size (Jin *et al.* 2001; Ranz *et al.* 2003; McIntyre *et al.* 2006; Wayne *et al.* 2007). McIntyre *et al.* (2006) analyzed 10,014 transcripts in eight lines of *D. melanogaster* and identified 5221 sex-biased transcripts at FDR 0.05 (56% male bias and 44% female bias). The overall sex effect for eight genotypes was 53%. Similarly, on a study using nine *D. melanogaster* lines, Wayne *et al.* (2007) reported 7617 out of 9312 genes with sexually dimorphic expression (4070 male bias and 3547 female bias). These previous studies used multiple genotypes of *D. melanogaster*. When the data for Wayne *et al.* (2007) were reanalyzed for each genotype separately, the percentage of sex-biased genes ranged from 1.29 to 40.09%. For the genotype considered in this study, 31% of genes showed a significant sex effect, close to the upper end of the range. A slight excess of genes with increased expression in females was also observed in this analysis, as is seen in previous analyses (Ranz *et al.* 2003). These findings are consistent with findings from arrays with a larger feature size.

Two previous studies using array designs (McIntyre *et al.* 2006; Telonis-Scott *et al.* 2008) found significant sex differences in alternative exon usage for many genes. For the single genotype examined here, approximately 5.6% of the genes examined showed evidence of sex-specific isoform expression. For four genes that are components of the sex determination pathway with previously reported sex-specific splicing in adults (*tra2*, *Sxl*, *dsx*, and *fru*), at least one exon shows evidence of sex bias.

This is the first genome-wide study of allele-specific expression variation within *D. simulans*. Although only one genotype was used, significant differences in AI were detected for 37% of probe sets examined that contained heterozygous SNP loci. Other work using *a priori* selected genes found almost 67% of the genes tested showed evidence for AI within species (Wittkopp *et al.* 2008). This chip can be used to detect allele-specific variation in expression within species. Large differences between males and females were detected in the number of probe sets that showed significant differences in AI. It is currently unclear whether this result is explained by differences in AI between males and females or by sex bias in overall expression making power for detection uneven.

As in microarray studies, to adequately assess ASE for a particular transcript using RNA-Seq, there must be adequate coverage for both alleles of that particular gene. For samples from the same organism and tissue, detection of transcription for a particular exon in four gigabases of RNA-Seq data are 57% (r. Graze *et al.*, unpublished data), whereas detection is 72% for tiling arrays (Graze *et al.* 2009). Initial studies of AI examined how many informative reads were needed per gene for estimation of allelic frequencies (Fontanillas *et al.* 2010). This study suggests that average depth of coverage needed is quite large if most genes are to be evaluated. The actual coverage needed depends on specific assumptions and the number varies, but it is often in excess of 100×. Other examinations of RNA-Seq find that a minimum average depth of five reads per nucleotide are needed for estimation of expression (McIntyre *et al.* 2011). One lane of a GAIIX provides sufficient reads at a coverage of 5× to assess ∼30% of the transcriptome (McIntyre *et al.* 2011). Arrays still provide a cost effective way of assessing transcription for the whole genome (Malone and Oliver 2011).

Detailed studies within species that examine AI variation genome-wide and identify the impact of sex on this variation are needed to understand the true extent of *cis* regulatory variation within species in *Drosophila*. This array is a good tool for such studies because it will allow the overall and allele-specific components of expression variation to be examined in a single experiment on a single platform for many more genes than has previously been possible.

## Supplementary Material

Supporting Information
