# Supplementary material for: Partitioning Transcript Variation in Drosophila: Abundance, Isoforms, and Alleles
Source: G3 (Bethesda). 2011 Nov 1;1(6):427–36. doi: 10.1534/g3.111.000596 (PMC3276160; doi:10.1534/g3.111.000596)
Supplement: Supporting Information [file supp_1_6_427__index.html]

Supporting Information 

# Partitioning Transcript Variation in *Drosophila*: Abundance, Isoforms, and Alleles

## Supporting Infomation for Yang *et al.*, 2011

**Files in this Data Supplement:**

- Supporting Information - Figures S1 and S2 and Tables S1-S5 (PDF, 688 KB)
- Figure S1 - Box plot of the signal intensity for 3� expression module probe sets (PDF, 80 KB)
- Figure S2 - Bland-Altman plots for gene means across modules in RNA arrays (PDF, 520 KB)
- Table S4 - Comparison between hybridization signal and sequence information (heterozygous genotypes) (PDF, 40 KB)
- Table S1 - Test results for sex effect in the expression module (.csv, 1.2 MB)
- Table S2 - Test results for alternative exon usage (.csv, 72 KB)
- Table S3 - Test results for sex effect in the exon module, probe sets corresponding to constitutive and non-overlapping exons only (.csv, 2.9 MB)
- Table S5 - Test result for AI in the SNP module, for the subset of unambiguous and heterozygous F1 genotype only (.csv, 1.3 MB)
