# Supplementary material for: Partitioning Transcript Variation in Drosophila: Abundance, Isoforms, and Alleles
Source: G3 (Bethesda). 2011 Nov 1;1(6):427–36. doi: 10.1534/g3.111.000596 (PMC3276160; doi:10.1534/g3.111.000596)
Supplement: Supporting Information [file supp_1.6.427_000596SI.pdf]

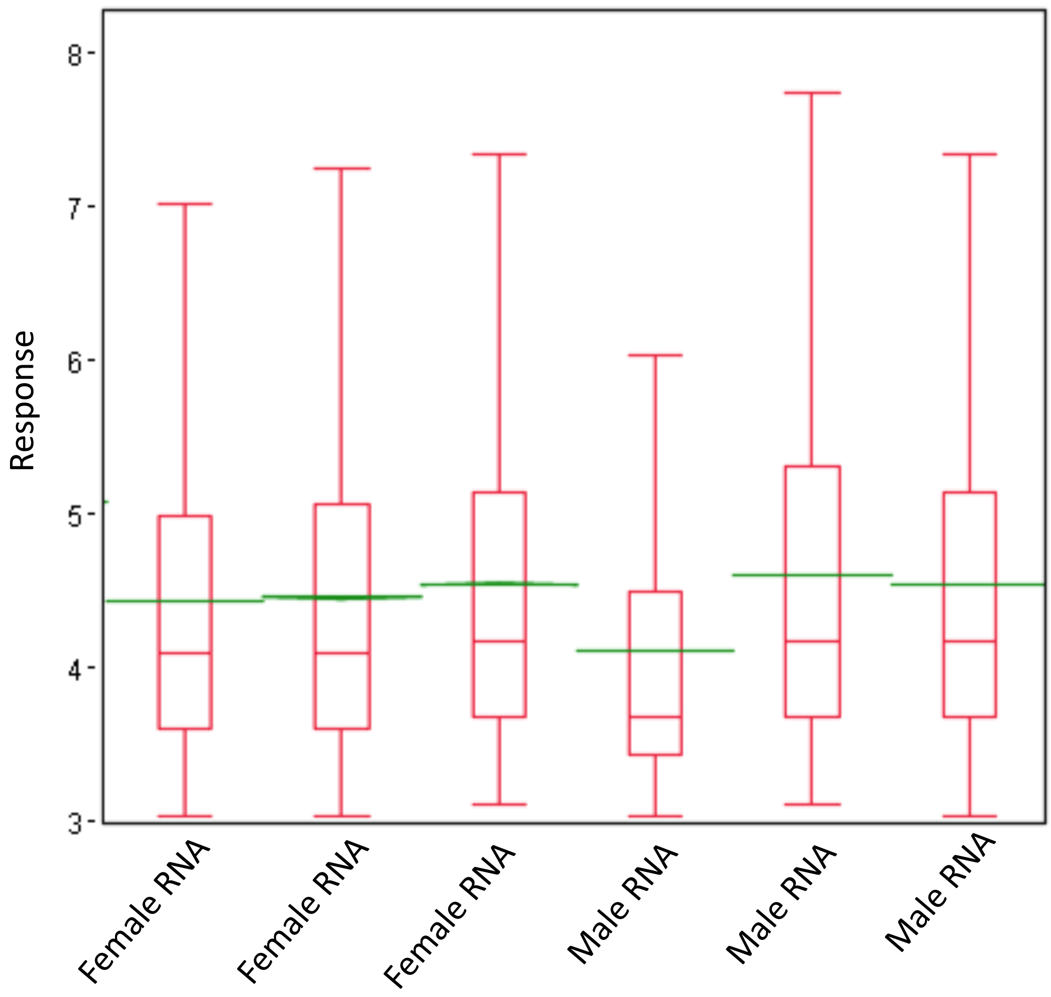

Figure S1 Box plot of the signal intensity for 3′ expression module probe sets. The three technical replicates for female RNA and for male RNA slides are shown separately. The y axis is the normalized signal. Means intensities are shown in green. The two sexes had similar overall hybridization patterns.

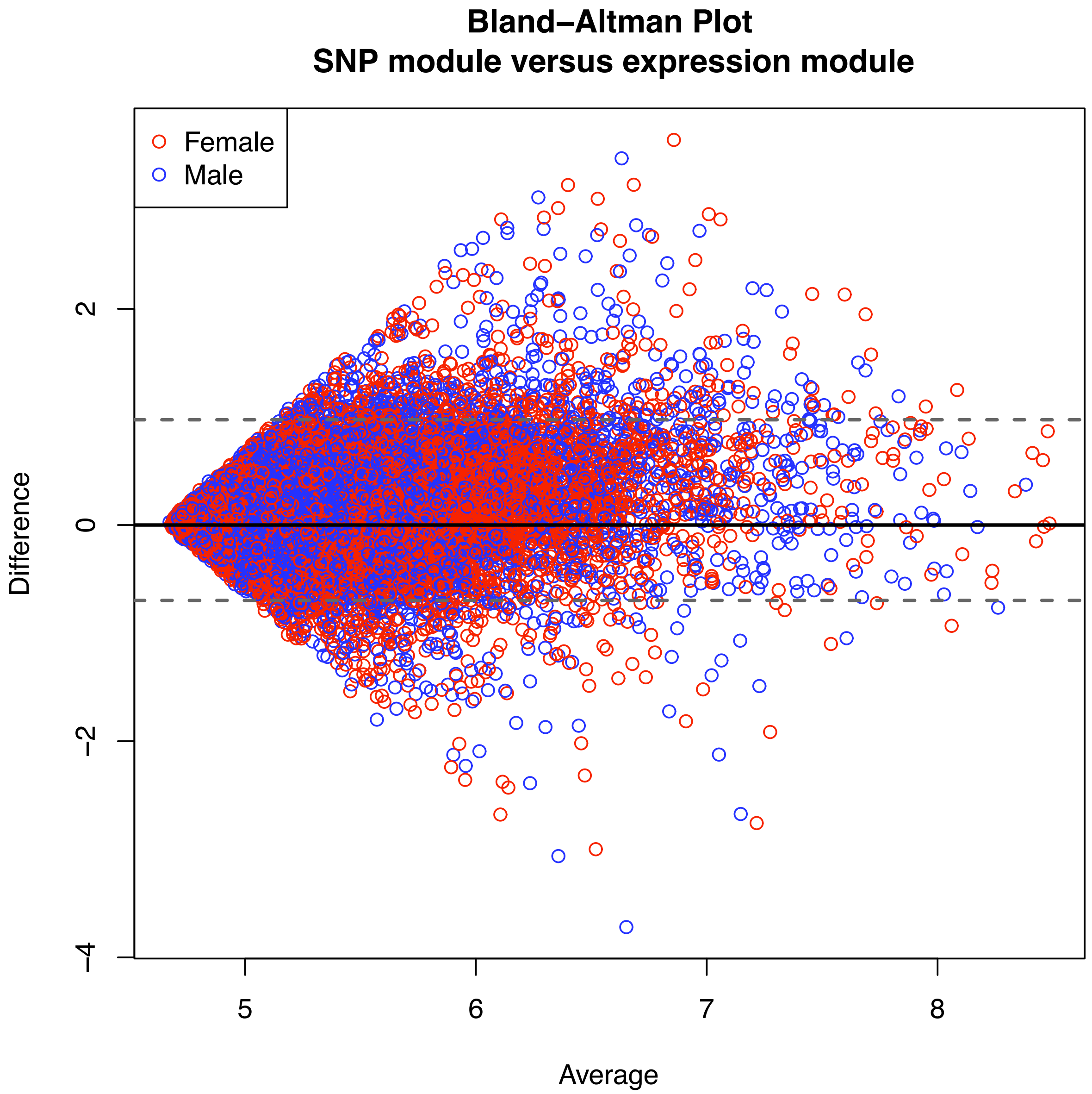
Bland–Altman Plot
SNP module versus expression module
Female
Male
Difference
Average
2
0
−2
−4
5
6
7
8

A

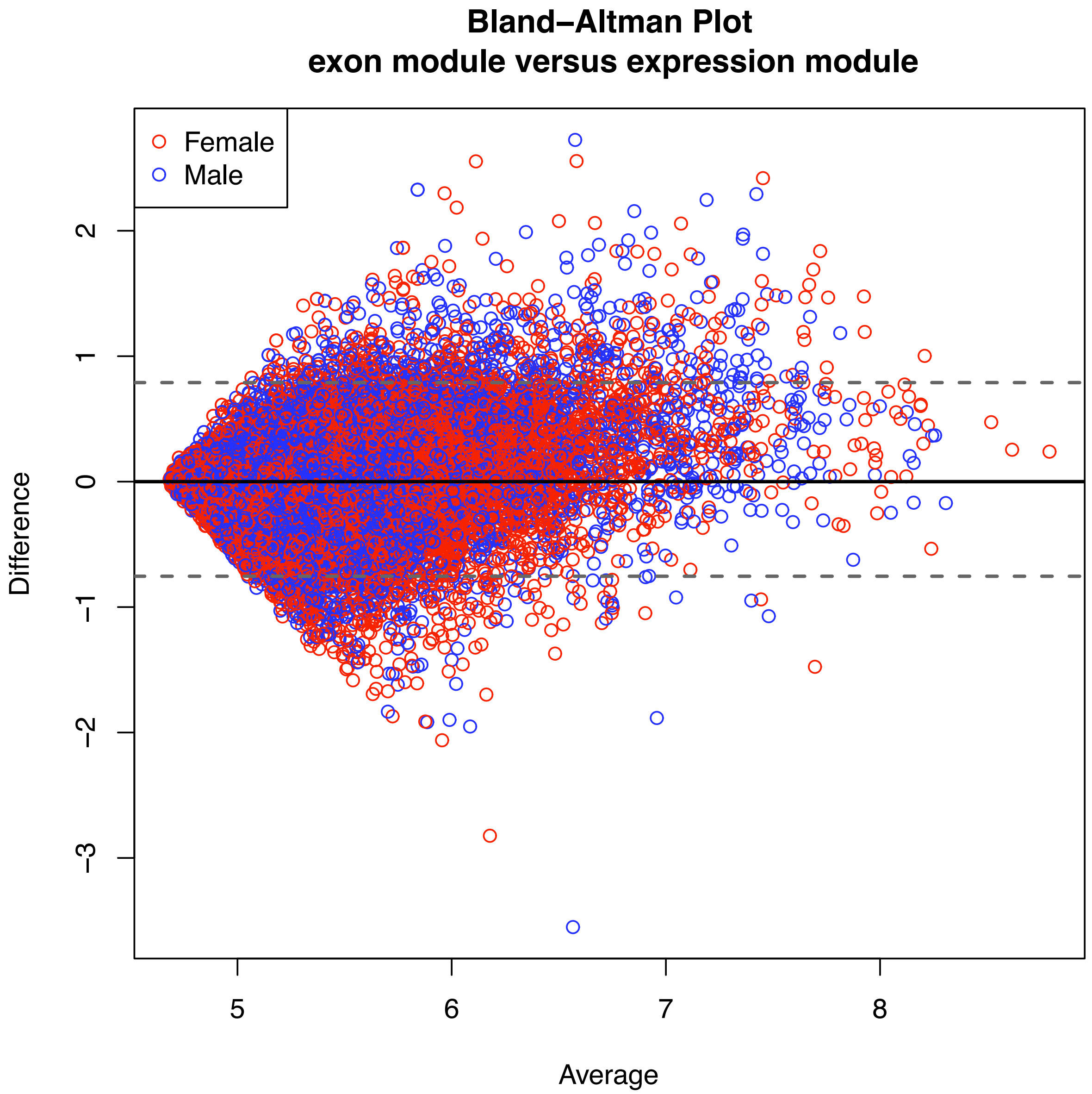

B

Figure S2 Bland-Altman plots for gene means across modules in RNA arrays.
The mean expression for each gene were calculated for the exon, 3' expression and SNP modules. Bland-Altman plots were cosntructed by plotting the average expression on the Y axis and the difference in expression on the X axis. Female samples are shown in red. Male samples are shown in blue. A. The SNP module compared to the 3' expression module. B. The exon module compared to the 3' expression module.

Table S1 Test results for sex effect in the expression module

Table S1 is available for download at http://www.g3journal.org/lookup/suppl/doi:10.1534/g3.111.000596/-/DC1 as a .csv file.

The results for tests of sex bias for the 3’ expression module. Probe set ID, raw P values, adjusted P values, binary flag for significance at FDR<0.05, binary flag for significance at FDR<0.1, binary flag for significance at FDR<0.2, expression mean of female samples, expression mean of male samples, and binary flag for whether the expression is biased toward females are reported.

Table S2 Test results for alternative exon usage

Table S2 is available for download at http://www.g3journal.org/lookup/suppl/doi:10.1534/g3.111.000596/-/DC1 as a .csv file.

The results for the tests of sex effect for the exon module. Gene symbol, raw P values, adjusted P values, binary flag for significance at FDR<0.05, binary flag for significance at FDR<0.1, and binary flag for significance at FDR<0.2, are reported.

Table S3 Test results for sex effect in the exon module, probe sets corresponding to constitutive and non-overlapping exons only

Table S3 is available for download at http://www.g3journal.org/lookup/suppl/doi:10.1534/g3.111.000596/-/DC1 as a .csv file.

Probe set ID, raw P values, adjusted P values, binary flag for significance at FDR<0.05, binary flag for significance at FDR<0.1, binary flag for significance at FDR<0.2, expression mean of female samples, expression mean of male samples, and binary flag for whether the expression is biased toward females are reported.

Table S4 Comparison between hybridization signal and sequence information (heterozygous genotypes)

Strain	SNP pair	Hybridized arrays			
		C167.4<br>DNA	*st e*<br>DNA	C167.4/*st e*<br>DNA	C167.4/*st e*<br>RNA
C167.4	A	56.68%	-	24.64%	29.31%
	C	78.27%	-	62.53%	71.20%
	G	79.33%	-	64.36%	71.78%
	T	57.46%	-	27.46%	31.86%
st e	A	-	85.53%	23.48%	20.18%
	C	-	95.43%	60.28%	62.39%
	G	-	95.38%	64.79%	66.50%
	T	-	84.40%	22.97%	18.26%

The table shows detailed comparison between hybridization signal and sequence information in heterozygous genotypes of F_1 (n=2,769). The percent of probe sets where the probes corresponding to the target SNP alleles corresponds to the top rank (parental genotypes) or the top two ranks (F_1 genotype) is reported separately for each base.

Table S5 Test result for AI in the SNP module, for the subset of unambiguous and heterozygous F1 genotype only

Table S5 is available for download at http://www.g3journal.org/lookup/suppl/doi:10.1534/g3.111.000596/-/DC1 as a .csv file.

AI was tested for the subset of unambiguous and heterozygous F1 genotypes only (refer the main text for subset details). Columns in this table are: probe set ID, mean signal of the mismatch (MM) probes for DNA samples, mean signal of the first positive match (PM) probes for DNA samples, mean signal for the second PM probes for DNA samples, mean signal of MM probes for female RNA samples, mean signal of PM1 probes for female RNA samples, mean signal of PM2 probes for female RNA samples, mean signal of MM probes for male RNA samples, mean signal of PM1 probes for male RNA samples, mean signal of PM2 probes for male RNA samples, raw P values for overall AI effect, adjusted P values for overall AI effect, binary flag for significance at FDR<0.05 for overall AI effect, binary flag for significance at FDR<0.1 for overall AI effect, binary flag for significance at FDR<0.2 for overall AI effect, raw P values for AI effect in female samples, adjusted P values for AI effect in female samples, binary flag for significance at FDR<0.05 for AI effect in female samples, binary flag for significance at FDR<0.1 for AI effect in female samples, binary flag for significance at FDR<0.2 for AI effect in female samples, raw P values for AI effect in male samples, adjusted P values for AI effect in male samples, binary flag for significance at FDR<0.05 for AI effect in male sampels, binary flag for significance at FDR<0.1 for AI effect in male samples, binary flag for significance at FDR<0.2 for AI effect in male samples, and binary flag for whether the expression is biased toward females.
