# Supplementary material for: Partitioning Transcript Variation in Drosophila: Abundance, Isoforms, and Alleles
Source: G3 (Bethesda). 2011 Nov 1;1(6):427–36. doi: 10.1534/g3.111.000596 (PMC3276160; doi:10.1534/g3.111.000596)
Supplement: Supporting Information [file supp_1.6.427_FigureS1.pdf]

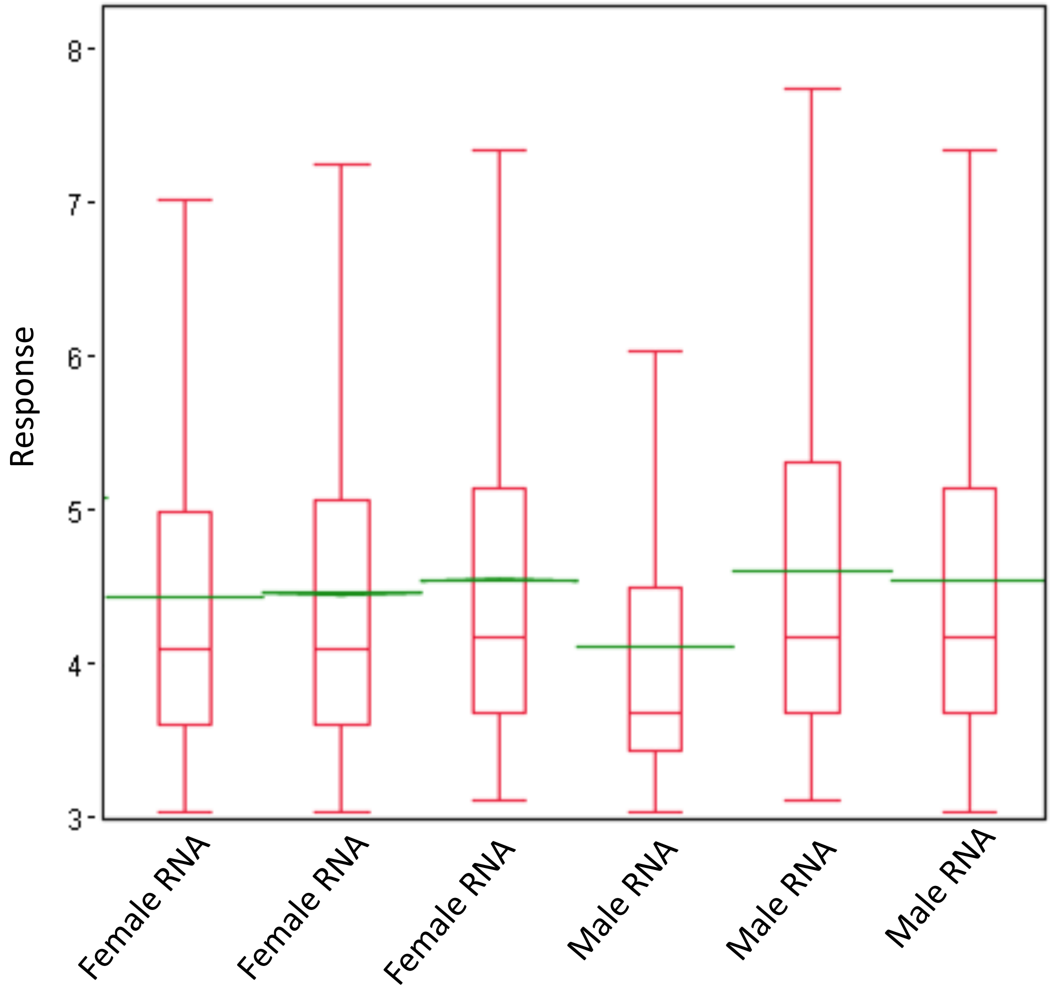

Figure S1 Box plot of the signal intensity for 3’ expression module probe sets. The three technical replicates for female RNA and for male RNA slides are shown separately. The y axis is the normalized signal. Means intensities are shown in green. The two sexes had similar overall hybridization patterns.
