# Supplementary material for: Partitioning Transcript Variation in Drosophila: Abundance, Isoforms, and Alleles
Source: G3 (Bethesda). 2011 Nov 1;1(6):427–36. doi: 10.1534/g3.111.000596 (PMC3276160; doi:10.1534/g3.111.000596)
Supplement: Supporting Information [file supp_1.6.427_FigureS2.pdf]

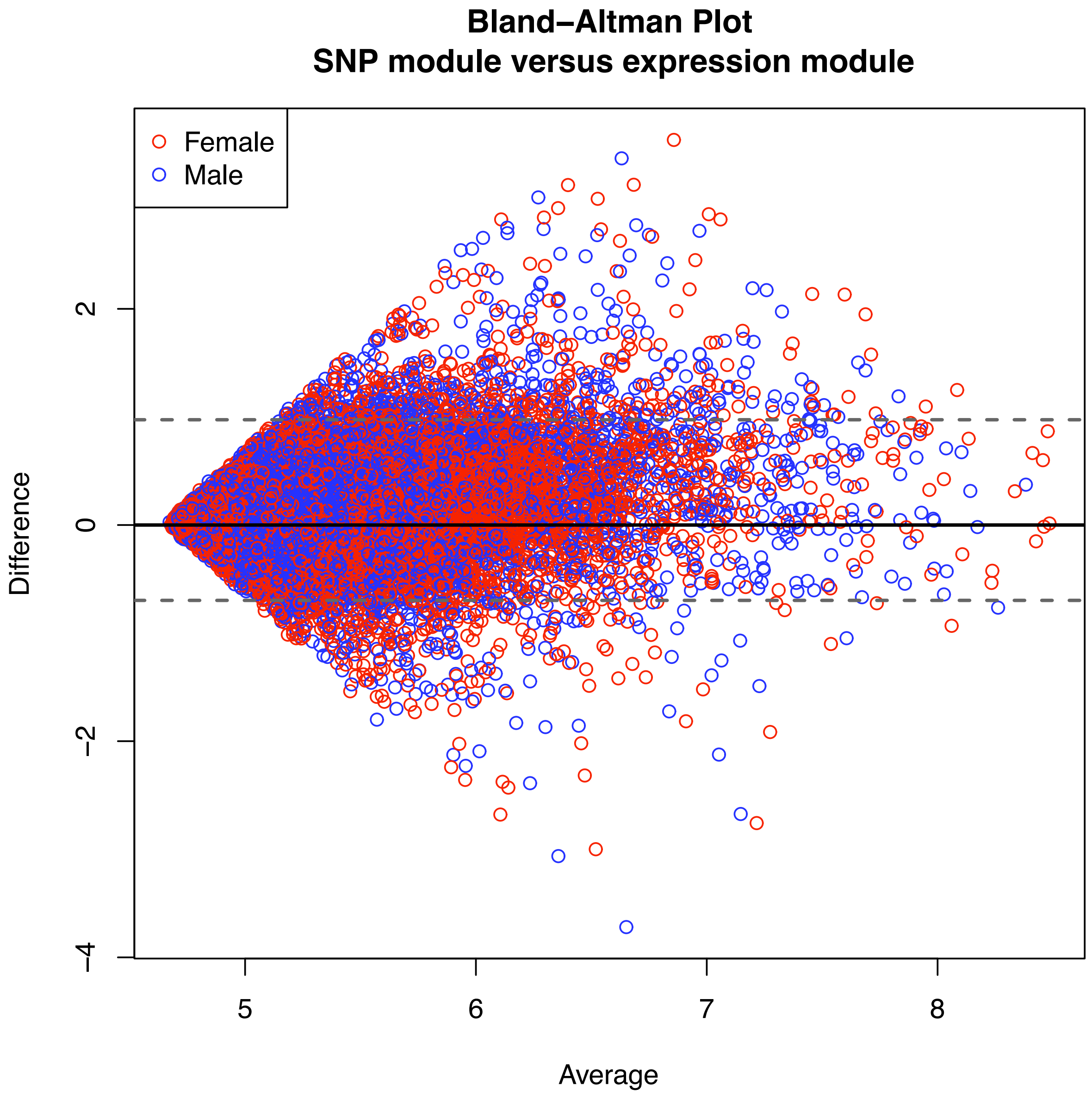
Bland–Altman Plot
SNP module versus expression module
Female
Male
Difference
Average
2
0
−2
−4
5
6
7
8

A

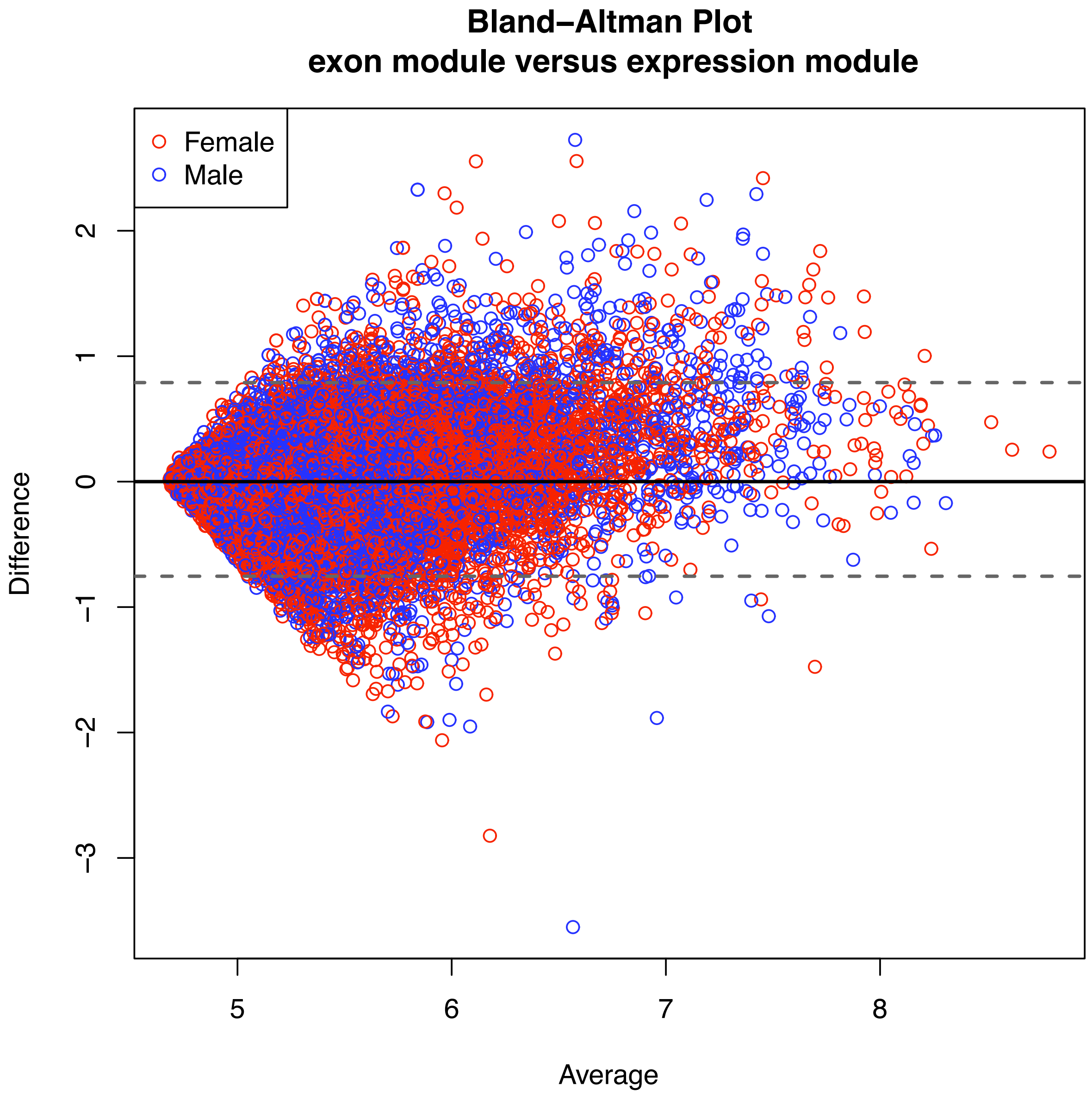

B

Figure S2 Bland-Altman plots for gene means across modules in RNA arrays.
The mean expression for each gene were calculated for the exon, 3' expression and SNP modules. Bland-Altman plots were cosntructed by plotting the average expression on the Y axis and the difference in expression on the X axis. Female samples are shown in red. Male samples are shown in blue. A. The SNP module compared to the 3' expression module. B. The exon module compared to the 3' expression module.
