# Supplementary material for: Partitioning Transcript Variation in Drosophila: Abundance, Isoforms, and Alleles
Source: G3 (Bethesda). 2011 Nov 1;1(6):427–36. doi: 10.1534/g3.111.000596 (PMC3276160; doi:10.1534/g3.111.000596)
Supplement: Supporting Information [file supp_1.6.427_TableS4.pdf]

Table S4 Comparison between hybridization signal and sequence information (heterozygous genotypes)

Strain	SNP pair	Hybridized arrays			
		C167.4 DNA	*st e* DNA	C167.4/*st e* DNA	C167.4/*st e* RNA
C167.4	A	56.68%	-	24.64%	29.31%
	C	78.27%	-	62.53%	71.20%
	G	79.33%	-	64.36%	71.78%
	T	57.46%	-	27.46%	31.86%
st e	A	-	85.53%	23.48%	20.18%
	C	-	95.43%	60.28%	62.39%
	G	-	95.38%	64.79%	66.50%
	T	-	84.40%	22.97%	18.26%

The table shows detailed comparison between hybridization signal and sequence information in heterozygous genotypes of F_1 (n=2,769). The percent of probe sets where the probes corresponding to the target SNP alleles corresponds to the top rank (parental genotypes) or the top two ranks (F_1 genotype) is reported separately for each base.
